# Predicting Mood Disturbance Severity with Mobile Phone Keystroke Metadata: A BiAffect Digital Phenotyping Study

**DOI:** 10.2196/jmir.9775

**Published:** 2018-07-20

**Authors:** John Zulueta, Andrea Piscitello, Mladen Rasic, Rebecca Easter, Pallavi Babu, Scott A Langenecker, Melvin McInnis, Olusola Ajilore, Peter C Nelson, Kelly Ryan, Alex Leow

**Affiliations:** ^1^ University of Illinois at Chicago Chicago, IL United States; ^2^ University of Michigan Ann Arbor, MI United States

**Keywords:** digital phenotype, mHealth, ecological momentary assessment, keystroke dynamics, bipolar disorder, depression, mania, mobile phone

## Abstract

**Background:**

Mood disorders are common and associated with significant morbidity and mortality. Better tools are needed for their diagnosis and treatment. Deeper phenotypic understanding of these disorders is integral to the development of such tools. This study is the first effort to use passively collected mobile phone keyboard activity to build deep digital phenotypes of depression and mania.

**Objective:**

The objective of our study was to investigate the relationship between mobile phone keyboard activity and mood disturbance in subjects with bipolar disorders and to demonstrate the feasibility of using passively collected mobile phone keyboard metadata features to predict manic and depressive signs and symptoms as measured via clinician-administered rating scales.

**Methods:**

Using a within-subject design of 8 weeks, subjects were provided a mobile phone loaded with a customized keyboard that passively collected keystroke metadata. Subjects were administered the Hamilton Depression Rating Scale (HDRS) and Young Mania Rating Scale (YMRS) weekly. Linear mixed-effects models were created to predict HDRS and YMRS scores. The total number of keystrokes was 626,641, with a weekly average of 9791 (7861), and that of accelerometer readings was 6,660,890, with a weekly average 104,076 (68,912).

**Results:**

A statistically significant mixed-effects regression model for the prediction of HDRS-17 item scores was created: conditional *R*^2^=.63, *P*=.01. A mixed-effects regression model for YMRS scores showed the variance accounted for by random effect was zero, and so an ordinary least squares linear regression model was created: *R*^2^=.34, *P*=.001. Multiple significant variables were demonstrated for each measure.

**Conclusions:**

Mood states in bipolar disorder appear to correlate with specific changes in mobile phone usage. The creation of these models provides evidence for the feasibility of using passively collected keyboard metadata to detect and monitor mood disturbances.

## Introduction

The burden of mental illness is high. It has been estimated that mental illness accounts for 32% of years lived with disability around the world [1]. Bipolar disorder is a serious mental illness characterized by recurrent episodes of depression and mood elevation [2] and is associated with high rates of functional impairment, decreased quality of life, and increased rates of mortality from comorbid medical conditions [3]. Given these costs, it is imperative that we deepen our understanding of this disorder to promote accurate diagnosis and effective treatment.

The ubiquity of mobile phones, smartphones in particular, presents a new opportunity in the study of mental illness. An estimated 64% of adults in the United States own a mobile phone and use it for a variety of tasks, including phone calls, Web browsing, and social media; however, the most widely and frequently used feature on mobile phones is short message service text messaging [4]. These devices can be employed as platforms for the unobtrusive collection of myriad data that can be used in the study of psychopathology. Ecological momentary assessment is a methodology that aims to collect data using repeated measures in real time (or near real time), in people’s natural environment [5]. When applied to the use of digital technologies such as mobile phones, this methodology can be used to create digital phenotypes defined as the set of observable behaviors resulting from the interaction between human disease and people’s use of digital technologies [6].

Because recurring mood episodes are a defining characteristic of bipolar disorder, we posited that it is an ideal illness for a pilot study investigating the relationship between mobile phone keyboard activity and the correlates of these episodes, such as changes in cognitive function, psychomotor activity, social behavior, and diurnal activity patterns. We elected to focus on keystroke dynamics because features using text input (eg, texting and Web browsing) are among the most commonly used features in mobile phones and because we hypothesized that keystroke dynamics provide a sufficiently dense space from which to extract relevant features that could be used to predict the severity of depression and mania.

## Methods

### Participants

Study subjects were members of the Prechter Longitudinal Study of Bipolar Disorder, a naturalistic, longitudinal study based in the University of Michigan [7]. This cohort includes subjects with bipolar disorder, other psychiatric illnesses, and healthy controls; however, only those with bipolar disorder were recruited into this study. Subjects were recruited into this study by email or phone invitation. The inclusion criteria included being a current Android mobile phone user, asserting familiarity with the Android operating system, having no gross impairments in fine motor abilities, sufficient vision to use a mobile phone keyboard, and self-reporting of frequent mood fluctuations or having longitudinal data from the longitudinal study suggesting that they experience frequent mood symptoms (ie, endorsed frequent mood symptoms on bimonthly self-report measures of mood or categorized as rapid cycling).

We initially included 19 subjects with a bipolar spectrum disorder as per the Diagnostic and Statistical Manual of Mental Disorders-Fourth Edition (Text Revision) criteria [8] (11 with bipolar I, 7 with bipolar II, and 1 with bipolar not otherwise specified); of these subjects, 1 never activated the app and 2 deleted the app early in the study. Of the remaining 16 subjects, participation varied in terms of the number of weeks that had any keyboard activity, with an average of 4.69 (3.05) weeks. Because of concerns about adherence, data analysis was restricted to subjects who provided data for at least 4 weeks. This resulted in 9 subjects: 5 with bipolar I and 4 with bipolar II. Of these, 8 subjects met the criteria for rapid cycling (ie, 4 or more mood episodes per year), and all subjects with bipolar II had recurrent depressive episodes. Of these 9 subjects, 7 showed keyboard activity for at least 6 weeks. The total usable data from these subjects included 626,641 keystrokes and 6,660,890 accelerometer readings.

### Mobile Keyboard

**Figure 1:**
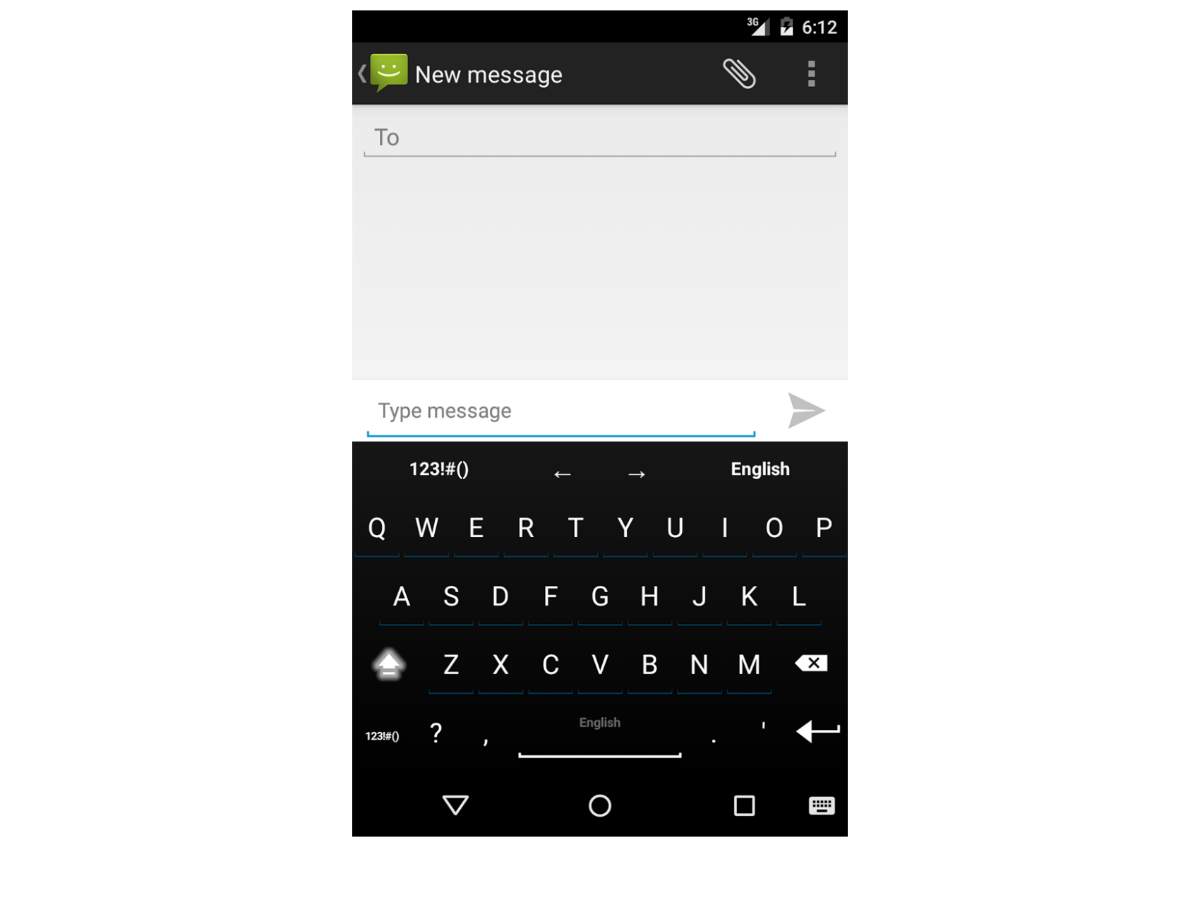
Screenshot of the BiAffect keyboard (keyboard design derived from AnySoftKeyboard by Menny Evan-Danan and licensed under Apache License 2.0.).

A custom keyboard called “BiAffect” was developed for the Android operating system that replaced the default keyboard and collected metadata consisting of keystroke entry date and time and accelerometer displacement. It uploaded these data using secure encrypted protocols to the study server hosted at the University of Illinois at Chicago. Accelerometer data collection was initiated by keystroke entry and continued for 5 seconds afterward. Individual character data outside of the backspace key and space bar were not collected, anonymizing the entry. The keyboard was designed to appear similar to the standard Android keyboard (Figure 1).

### Data Collection

For 8 weeks, subjects were provided a Samsung Galaxy Note 4 smartphone that they were instructed to use as their primary phone during the study period. Subjects were encouraged to use their current phone number and subscriber identification module card; with the exception of 1 subject, all subjects did so. During the study period, trained staff at the University of Michigan administered the Structure Interview Guide for the Hamilton Depression Rating Scale (HDRS) [9] and Young Mania Rating Scale (YMRS) [10] once a week via phone interviews.

### Statistical Analyses

Subject demographics are described in Table 1. The YMRS results showed a right-tailed skew (*γ*_1_=1.14) [11], so a log transformation was performed on the YMRS scores by taking the natural log of the sum of the YMRS scores and 1 (*γ*_1_=−0.44).

In order to identify the possible relationships between subject demographics and phone usage, Spearman correlations were calculated between subjects’ total key counts and their age and education.

Mixed-effects linear models were created correlating keyboard metadata collected from the week prior to the administration of the HDRS (17-item) and YMRS mood rating scores. Missing data were handled with pairwise deletion. Features extracted from the metadata were modeled as fixed effects. Observations were grouped by subject, with each subject having his or her own random intercept for his or her mood ratings.

**Table 1:** Subject characteristics.

Characteristics		Value	
Age in years, mean (SD)		48.67 (9.63)	
Female gender, n (%)		8 (89)	
Years of education, mean (SD)		14.00 (2.12)	
**Diagnosis, n (%)**			
	Bipolar I		1 (11)
	Bipolar I with rapid cycling		4 (44)
	Bipolar II, recurrent depressive episodes, with rapid cycling		4 (44)
Number of keystrokes, mean (SD)		69,627 (57,477)	
Number of accelerometer readings, mean (SD)		740,099 (47,165)	
Weeks of activity, mean (SD)		7.70 (0.70)	
Initial HDRS^a^-17 item, mean (SD)		11.90 (6.17)	
Final HDRS-17 item, mean (SD)		11.11 (5.49)	
Initial YMRS^b^, mean (SD)		7.56 (5.00)	
Final YMRS, mean (SD)		6.67 (4.03)	

^a^HDRS: Hamilton Depression Rating Scale.

^b^YMRS: Young Mania Rating Scale.

**Table 2:** Predictor variable definitions.

Predictor variable	Definition
Average interkey delay	The average time between keystrokes measured in seconds
Backspace ratio	Number of backspace keypresses divided by total keypresses
Autocorrect rate	Number of autocorrect events divided by total keypresses
Circadian baseline similarity	The cosine-based similarity between the hourly distribution of keypresses/week and the hourly distribution for the study period
Average accelerometer displacement	Square root of sum of squares of accelerometer displacement along each coordinate (x, y, z) averaged over the week (average of √x^2^+y^2^+z^2^)
Average session length	Length of sessions in seconds averaged over the week
Session count	Number of sessions: A session begins when a keypress is initiated and ≥5 s has elapsed since the last key was pressed. A session ends when ≥5 s has elapsed since the last key was pressed.

Overall significance was assessed by using likelihood ratio tests comparing the null models that consisted of just the subject-level effect with full models consisting of the subject-level effect and metadata features. Because the mixed-effects model for the YMRS scores showed that the random effect was accounting for none of the variance of the YMRS scores, a fixed-effects ordinary least squares model was created instead (mixed-effects model log likelihood −64.621, Akaike Information Criterion 149.24, Bayesian Information Criterion 170.83; fixed-effects ordinary least squares model: log likelihood −64.621, Akaike Information Criterion 147.24, Bayesian Information Criterion 166.67). For the HDRS model, conditional and marginal *R*^2^ values were calculated using the method specified by Nakagawa and Schielzeth [12], as implemented in the R package piecewiseSEM [13]. Using this method, the conditional *R*^2^ is equal to the proportion of variance explained by both the fixed and random effects, and the marginal *R*^2^ is equal to the proportion of the variance explained by the fixed effects alone. The *P* values of the model coefficients were calculated using Wald chi-square tests, as implemented in the R package car [14] for the HDRS model. For the YMRS model, overall significance was tested using an F-test and individual coefficient significance was determined with t-tests.

The fixed-effect variables included the average interkey typing delay, the average accelerometer displacement, the backspace and autocorrect rates (ie, the total number of each divided by the total number of keystrokes), the average length of each typing session in seconds, the total number of typing sessions, and the cosine similarity between each week’s keypress activity and the total keypress activity of the study period (described further below). All aggregate variables were calculated for the week preceding each mood assessment. A session was defined as beginning with a keypress that occurs after 5 or more seconds have elapsed since the last keypress and ending when 5 or more seconds have elapsed between keypresses.

Models were created using the software package lme4 [15] for the R software environment version 3.3.3 [16].

### Predictor Variables

The predictor variables were chosen based on the hypothesis that they map to key cognitive and behavioral domains affected by mania and depression. Table 2 provides definitions of each variable, and each domain and their corresponding variables are discussed in turn below.

#### Psychomotor Activity

As per the Diagnostic and Statistical Manual of Mental Disorders, Fifth Edition (DSM-5), changes in psychomotor functioning are criteria for both major depressive and manic episodes [2]. Psychomotor activity is also a component of the clinician’s ratings within HDRS and YMRS. We hypothesized that psychomotor activity (agitation and retardation) manifests in the accelerometer displacement and the average interkey delay. We predicted that increasing levels of psychomotor agitation lead to subjects holding their phones less stably, thus resulting in higher accelerometer displacement values. In the case of average interkey delay, it can be argued that increased levels of psychomotor agitation could lead to either a lower or higher delay. In the case of the former, higher levels of agitation would lead to a general speeding up of behaviors, including typing; however, it is also possible that while more agitation may lead to an increase in the amount of activity, the ability to effectively type will be impaired, leading to a higher interkey delay and possibly more use of backspace and autocorrect. In contrast, psychomotor retardation was hypothesized to manifest as a higher average delay.

#### Social Activity

The BiAffect app did not capture the context of keyboard activity; however, we hypothesized that increases in keyboard activity are likely associated with increased social activity consisting of both texting and social media usage and that more activity would be associated with higher YMRS scores and lower HDRS scores. There are mixed data on the role of social media use and depression, with some studies showing decreases [17] and others reporting increases in social media usage in both high school [18] and college [19] students.

#### Cognition

Impairments in attention and concentration are seen in both depressive and manic episodes, as described in the DSM-5 and previous studies [2]. Impulsivity and deficits in error correction have also been identified as features seen in manic episodes [20]. Variables that characterize concentration and cognition were hypothesized to include the average interkey delay, the backspace rate, and the autocorrect rate. It was hypothesized that increased backspace rates indicated increased error correction and increased autocorrect rates indicated decreased error detection. Impaired concentration was hypothesized to manifest as increased interkey delay.

#### Diurnal Activity Patterns

Changes in sleep patterns are characteristic of both depressive and manic episodes. In the case of depression, this may take the form of insomnia or hypersomnia, whereas in the case of mania, there is typically a decreased need for sleep [2]. We expected that such changes in sleeping patterns would manifest as changes in phone typing activity. To characterize such changes, we created a cosine-based similarity feature of keypress activity. Cosine-based similarity is a frequently used technique in the field of machine learning and predictive algorithms to characterize the similarity between entities [21,22]. In our implementation, the distribution of keypress activity for a given week was defined as vector of 24 dimensions, with each dimension corresponding to an hour of the day. The value of the vector in each dimension was set equal to the number of keypresses in that hour. We then calculated the cosine of the angle between each week’s vector and the vector representing activity for the entire study period. In this way, the more dissimilar a given week’s pattern of activity was compared to the total activity, the lower the value of the cosine would be. It was hypothesized that more dissimilar weeks would correspond to higher HDRS and YMRS scores.

## Results

### Predictor Variable Summary Statistics

Summary statistics for each predictor variable are presented in Table 3.

### Total Key Press Activity and Subject Demographics

No statistically significant correlations were found between total key counts and subjects’ age (S=139.16, *P*=.68) and education levels (S=144.41, *P*=.60).

### Prediction of Depression Symptoms

Likelihood ratio testing comparing the null model that consisted of just the subject-level random effect to the full model showed that the full model had superior fit (*χ* ²_7_=17.6, *P=*.01; see Tables 4 and 5). The marginal *R*^2^ (ie, the proportion of the variance explained by the metadata features) was 0.41, and the conditional *R*^2^ (ie, the proportion of the variance explained by both the subject-level effect and the metadata features) was .63. Accelerometer displacement (*P=*.002), average interkey delay (*P=*.02), session count (*P=*.003), and the autocorrect rate (*P=*.004) were found to be positively correlated with the HDRS scores.

### Prediction of Hypomania or Mania Symptoms

A multiple linear regression model was created that accounted for 34% of the variance of the natural logarithm of YMRS scores (multiple *R*^2^=.34, *F*_7,56_=4.08, root mean square error=.66, *P=*.001; Table 5). Accelerometer displacement (*P=*.003) was found to be positively correlated with YMRS scores, and the backspace rate (*P=*.01) was found to be negatively correlated.

**Table 3:** Variable summary statistics.

Statistics	Mean (SD)	Min	Max	Average number of observations per subject per week
Average accelerometer displacement (m/s^2^)	9.56 (0.22)	9.07	9.99	104,076
Average interkey delay (s)	0.69 (0.36)	0.30	1.95	9780
Backspace rate	0.093 (0.050)	0.0070	0.27	982^a^
Autocorrect rate	0.10 (0.033)	0.011	0.14	1021^a^
Average session length (s)	19.63 (6.12)	6.90	31.51	—
Session count	241.13 (159.33)	9	804	—
Circadian baseline similarity	0.77 (0.17)	0.14	0.96	—
HDRS^b^ 17-item	11.83 (6.29)	0	25	—
YMRS^c^	5.64 (4.87)	0	20	—
Natural log of YMRS	1.60 (0.82)	0	3.045	—

^a^Average number of backspace or autocorrect events.

^b^HDRS: Hamilton Depression Rating Scale.

^c^YMRS: Young Mania Rating Scale.

**Table 4:** Fixed effects estimates of regression models.

Scaled predictors	17-item Hamilton Depression Rating Scale		Natural log of Young Mania Rating Scale	
	Linear mixed-effects (95% CI)	*P* value	Ordinary least squares (95% CI)	*P* value
Average accelerometer displacement	3.20 (1.20 to 5.21)	.0017	0.39 (0.15 to 0.64)	.003
Average interkey delay	2.88 (0.42 to 5.35)	.022	0.13 (−0.19 to 0.44)	.44
Backspace ratio	−0.01 (−1.53 to 1.52)	.99	−0.30 (−0.53 to −0.070)	.014
Autocorrect rate	2.67 (0.87 to 4.47)	.0036	0.06 (−0.17 to 0.29)	.63
Average session length	−1.16 (−2.71 to 0.39)	.14	−0.04 (−0.24 to 0.16)	.68
Session count	2.18 (0.77 to 3.56)	.0025	−0.04 (−0.28 to 0.19)	.73
Circadian baseline similarity	0.34 (−1.07 to 1.75)	.64	0.03 (−0.22 to 0.27)	.83
Constant	11.77 (9.80 to 13.74)	<.001	1.60 (1.43 to 1.78)	<.001

**Table 5:** Summary of regression results.

Scaled predictors	17-item Hamilton Depression Rating Scale	Natural log of Young Mania Rating Scale
	Linear mixed-effects	Ordinary least squares
Observations	64	64
Multiple *R*^2^	—	.34
Adjusted *R*^2^	—	.26
Conditional *R*^2^	.63	—
Marginal *R*^2^	.41	—
Log likelihood	−179.65	—
Residual standard error	—	.71^a^
Chi-square statistic or F statistic	17.6^b^	4.1^c^

^a^*df*=56

^b^*χ*^2^_7_, *P*=.014.

^c^*F*_7,56_, *P*=.0011.

## Discussion

### Principal Findings

Using only passively collected metadata, keystroke activity predicted both depressive and manic symptoms. The model to predict depression scores demonstrated greater explanatory capacity as shown by the larger proportion of variance explained by the model and the larger number of significant predictors.

### Psychomotor Activity

Increased accelerometer activity was found to be positively correlated with both depression and mania scores. One possible explanation for the positive correlation with both scores is that the subjects in our study had more mildly agitated or irritable forms of depression or depression with mixed features rather than forms exhibiting psychomotor retardation.

### Social Activity

In contrast to our hypothesis that decreased sessions would be predictive of higher depression, the overall number of sessions was actually positively correlated with depression. This may be a reflection of the dynamic between loneliness and withdrawal. Sessions from a phone can be seen as lower risk and can also include passive use of social media, such as viewing but not posting, enabling a feeling of connection and withdrawal. At least one study has demonstrated an association between increased usage of the internet more generally and depressive symptoms [23]. It is also worth noting that while the session count was positively correlated, the average session length was negatively correlated (although this predictor did not reach statistical significance, *P=*.15), suggesting that patterns of activity may be more relevant than the overall volume of activity.

### Cognition

Impairments in executive function have been demonstrated more in individuals with bipolar disorder in depressed, manic, and euthymic states than in healthy controls [20], although it has also been shown that executive functioning may be especially impacted during manic states [24,25]. Interestingly, our depression and mania symptom models diverge in their relationships with respect to what we theorized would be the key features related to cognition: backspace and autocorrect rates. The increase in autocorrect rate with depression symptoms seems relatively straightforward. Here, the ability to concentrate becomes impaired in more depressed states, and therefore, the rate of typing errors increases. What is less clear is why the backspace rate would be negatively correlated with mania symptoms without a concomitant positive correlation with the autocorrect rate. One possibility is that the lower backspace usage seen with higher mania scores reflects a phenomenon of less self-monitoring or impaired response inhibition with errors. Those with elevated mania do not trigger the autocorrect mechanism because their inputs are generally correctly spelled but often grammatically or semantically inappropriate words, fitting the profile of someone who keeps deleting what they type because it was impulsively entered.

### Diurnal Activity Patterns

Because sleep disturbance is such a prominent aspect of mood disturbance, we were surprised that measurements that aimed to reflect diurnal variations in activity were not predictive of depressive or mania symptoms. With the assumption that the distribution for the entire observation period would approximate the subject’s baseline, we expected that lower values of similarity would be correlated with higher depression and mania scores. The cosine similarity values did not reach statistical significance in both models. One possible explanation for this is that the period of observation was not long enough to establish actual baselines in the sense of encompassing activity through a variety of mood states, including euthymia, and that the distribution for the entire observation period for many subjects corresponded to a single mood state. Another important consideration is that while diurnal patterns of phone activity may be related to sleep, they are not identical.

### Limitations and Future Directions

The limitations of this study include its sample size (relative to the model’s complexity), sample characteristics that are probably not representative of a general population (ie, mostly women who have a high frequency of episodes), and the constraint of having subjects using study-issued phones. A larger study in which participants use their own phones is warranted in order to determine the generalizability of these findings. More data may also enable the creation of more sophisticated models with higher rates of prediction accuracy and reliability.

Unfortunately, there were fewer predictors of mania scores, and overall, this prediction was less accurate. Prediction of acute changes in mania may have stronger clinical implications, given the reduced tendency to seek treatment in mania generally. We suspect that primary reasons for the decreased prediction of mania are that our sample contained generally low mania scores and that both mania and hypomania elevations are often short and sporadically observed relative to longer and more stable episodes of depression. Rather than demonstrating correlates of mania per se, the mania model presented here might represent correlates of mixed or agitated depression.

### Comparison with Prior Work

Prior studies have investigated the potential utility of various aspects of mobile phone activity as a means to diagnose mood states. Early studies focused on demonstrating the practicality of collecting self-reports of mood using mobile phones from patients [26,27]. While this approach may increase the facility with which such data are collected, it is still subject to the biases associated with self-reported data, potentially leading to spurious results [28]. More recent studies have focused on the validation of passive data collection methods and yielded encouraging results. Passive data features that have been demonstrated to correlate with mood ratings include physical movement [29,30], amount of phone usage [30], and frequency of calls and text messages with personal contacts [31].

The use of keystroke dynamics as a means to detect the emotion or mood of users is an active area of research in the field of affective computing, with most studies to date investigating the use of desktop keyboards [32]; however, there have been at least two studies that have examined the use of mobile phone keyboard dynamics as means to recognize user emotion. The first study was a 2-week pilot study based on the activity of a single user on Twitter, wherein the user was instructed to write a Tweet whenever he or she experienced certain emotions and to record the emotion from a preset selection of options. Using a Bayesian Network classifier, the investigators were able to achieve an overall classification accuracy of 67.52%, with the most important feature being typing speed [33]. The second study consisted of a larger sample of 22 subjects and was conducted over 3 weeks. It also presented users with a preset selection of options for emotions; although, in contrast to the first study, keyboard activity was recorded over all applications and the users were prompted to input their emotional state on a regular basis. Using a random forest model, the investigators were able to achieve an average classification accuracy of 84%, with the most important typing dynamic feature being typing speed [34].

Although the aforementioned studies measuring mobile phone keystroke dynamics sought to predict emotion rather than mood, we find the relative importance of typing speed as an important feature across their studies as well as our own to be of note. To the best of our knowledge, our study is the first effort to use passively collected mobile phone keyboard metadata features to predict mood disturbances in a clinical sample using clinically relevant measures.

### Conclusions

Passively collected mobile phone keystroke dynamics may be a useful and important method to identify incipient mood processes in persons with bipolar disorder. The facility with which such data may be used to infer the presence and severity of mood disturbances may enable clinical providers to intervene earlier in their patients’ mood episodes, as well as increase the number of patients a single provider can effectively manage. Models such as those presented here may also lead to a deeper understanding of these disorders by revealing novel behavioral traits associated with them.

## Abbreviations

DSM-5: Diagnostic and Statistical Manual of Mental Disorders, Fifth Edition
HDRS: Hamilton Depression Rating Scale
YMRS: Young Mania Rating Scale
SMS: short messaging service
